# After the initial fracture in postmenopausal women, where do subsequent fractures occur?

**DOI:** 10.1016/j.eclinm.2021.100826

**Published:** 2021-05-05

**Authors:** Carolyn J. Crandall, Rebecca P. Hunt, Andrea Z. LaCroix, John A. Robbins, Jean Wactawski-Wende, Karen C. Johnson, Maryam Sattari, Katie L. Stone, Julie C. Weitlauf, Tanya R. Gure, Jane A. Cauley

**Affiliations:** a Department of Medicine, Division of General Internal Medicine and Health Services Research, David Geffen School of Medicine at the University of California, 1100 Glendon Ave. Suite 850 - Room 858, Los Angeles, CA 90024, United States; b WHI Clinical Coordinating Center, Fred Hutchinson Cancer Research Center, Seattle, WA, United States; c Herbert Wertheim School of Public Health and Human Longevity Science, University of California, La Jolla, San Diego, CA, United States; d Department of Medicine, UC Davis Medical Center, Sacramento, CA, United States; e Department of Epidemiology and Environmental Health, University at Buffalo the State University of New York, Buffalo, NY, United States; f Department of Preventive Medicine, University of Tennessee Health Science Center, Memphis, TN, United States; g Division of General Internal Medicine, University of Florida, Gainesville, FL, United States; h California Pacific Medical Center Research Institute, San Francisco Coordinating Center, San Francisco, CA, United States; i Veterans Affairs Palo Alto Health Care System, Palo Alto, CA, Department of Psychiatry and Behavioral Sciences, Stanford University, Stanford, CA, United States; j Division of General Internal Medicine & Geriatrics, The Ohio State University, Columbus, OH, United States; k Department of Epidemiology, Graduate School of Public Health, University of Pittsburgh, Pittsburgh, PA, United States

**Keywords:** Fracture, Osteoporosis

## Abstract

**Background:**

The locations of subsequent fractures after initial fracture in postmenopausal women are poorly characterized.

**Methods:**

We conducted a prospective analysis of subsequent fractures after initial fracture in Women's Health Initiative (1993–2018) participants who provided follow-up (mean 15.4 years, SD 6.2 years) data (*n* = 157,282 participants; baseline age 50–79; 47,458 participants with incident fracture). Cox proportional hazards models were adjusted for age, race/ethnicity, body mass index, and other covariates.

**Findings:**

The risk of each type of subsequent fracture was increased after each type of initial fracture. Incident lower arm/wrist fracture was associated with significantly elevated risks of subsequent fractures at the upper arm/shoulder, upper leg, knee, lower leg/ankle, hip/pelvis, and spine (adjusted hazard ratios [aHRs] ranging 2·63–5·68). The risk of hip fracture was increased after initial lower arm or wrist fracture (aHR 4·80, 95% CI 4·29–5·36), initial upper arm or shoulder fracture (aHR 5·06, 95% CI 4·39–5·82), initial upper leg fracture (aHR 5·11, 95% CI 3·91–6·67), initial knee fracture (aHR 5·03, 95% CI 4·20–6·03), initial lower leg/ankle fracture (aHR 4·10, 95% CI 3·58–4·68), and initial spine fracture (aHR 6·69, 95% CI 5·95–7·53). Associations were significant in all age groups, even women aged 50–59 years. Risks of subsequent fracture were more pronounced among non-Hispanic Black, Hispanic/Latina, and Asian/Pacific Islander than among non-Hispanic White women.

**Interpretation:**

Increased risk of subsequent fracture is observed for all fracture types across all ages. Women who experience any of these fractures should be targeted for interventions to prevent subsequent fractures.

**Funding:**

National Institutes of Health HHSN268201600018C,HHSN268201600001C, HHSN268201600002C, HHSN268201600003C, and HHSN268201600004C.

**Research in context**

**Evidence before this study**

Current clinical osteoporosis guidelines emphasize the risk of subsequent fracture following initial vertebral and hip fracture, but do not focus on other types of initial fractures. We searched PubMed (inception-1/4/2020) for original research reports using the search terms “secondary fracture”, “second fracture”, “location”, “initial fracture”, “subsequent fracture”, and “epidemiology”. Detailed information is lacking from prospective large cohort studies regarding anatomical locations of subsequent fracture after initial fracture among postmenopausal women in the U.S. This information would highlight the burden of potentially preventable fractures and inform the development of targeted secondary prevention strategies as well as clinical guidelines.

**Added value of this study**

Among postmenopausal women, every type of initial fracture (lower arm or wrist, upper arm or shoulder, upper leg, knee, lower leg, ankle, and hip or pelvis fracture) is associated with significantly increased risk of subsequent fracture. Moreover, the higher risk of subsequent fracture after initial fracture was pronounced in all age groups, even younger postmenopausal women aged 50 to 59 years. We identified important racial/ethnic differences; non-Hispanic Black women had markedly higher risk of subsequent fracture than did non-Hispanic White women.

**Implications of all the available evidence**

Clinicians should be aware that initial fractures of any type in postmenopausal women, even at sites other than the hip, vertebra, or wrist, should trigger counseling regarding increased subsequent fracture risk. Women of all ages, including younger women aged 50 to 59 years, who have initial fracture should be counselled that they are elevated risk of subsequent fracture. These results have also public health implications because the racial/ethnic differences in subsequent fracture risk were not previously recognized, potentially missing a large burden of preventable fractures in minority U.S. populations. Clinical guidelines should expand their risk assessment algorithms to include this information. Future research should examine potential reasons for the identified racial/ethnic differences.

Alt-text: Unlabelled box

## 1 Introduction

Currently, there is a crisis of under-treatment of osteoporosis [1,2], with a marked decline in the prescribing of oral and intravenous bisphosphonates in the U.S. between 2007 and 2012 [3]. The rates of initiation of osteoporosis medication within 6 months of hospitalization for hip fracture have declined over the past 15 years from 10 to 3% [4]. In 2017, only 3 in 10 fractures in the U.S. were followed up with bone density testing or treatment [5]. The under-treatment of osteoporosis is particularly important because one half of all postmenopausal women will have an osteoporosis-related fracture during their lifetimes, and because osteoporotic fractures are associated with chronic pain and disability, loss of independence, decreased quality of life, and increased mortality [6]. An estimated 12·3 million individuals older than 50 years in the United States have osteoporosis [7].

Some of the under-treatment of osteoporosis has been attributed to Food and Drug Administration announcements regarding potential risks of bisphosphonate therapy [8]. Another source of under-treatment may be under-recognition that persons with prior fracture are at high risk for future fracture. Twenty percent of women experience a subsequent fracture within 10 years of an initial wrist fracture [9]. However, detailed information from large prospective U.S. cohort studies regarding anatomical locations of subsequent fracture after initial fracture is lacking. This information would highlight the burden of potentially preventable fractures and inform the development and testing of targeted secondary prevention strategies.

The objectives of this study were to determine, among postmenopausal women aged 50 years and older, the incidence of subsequent fracture following an initial fracture during 10-year prospective follow-up, stratified by the anatomical site of initial fracture, age at initial fracture, and race/ethnicity.

## 2 Methods

### 2.1 Study participants

The Women's Health Initiative (WHI) enrolled postmenopausal women aged 50 to 79 years at 40 U.S. clinical centers between 1993 and 1998 [10]. Exclusion criteria included predicted survival time of less than 3 years or conditions or characteristics interfering with study participation and/or adherence (alcoholism, mental illness, dementia). The WHI Observational Study (WHI-OS) examined the predictors and natural course of important causes of morbidity and mortality in postmenopausal women. The three WHI Clinical Trials (WHI-CT) tested a low fat eating pattern, menopausal hormone therapy, and calcium plus vitamin D supplementation [10]. The main study was carried out between 1993 and 2005. Height and weight were measured at baseline. Body mass index (BMI) was calculated as body weight in kilograms (kg) divided by the square of height in meters (m). After the main study, WHI invited all participants to continue participation in the Extension Studies, Extension Study I (2005–2010), Extension Study II (2010–2015), and Extension Study III (2015-present). This study includes data from October 1993-March 31, 2018. CJC and RPH had access to the data, which were available between January 1, 2020 and March 18, 2021. Institutional review board approval was obtained at each center. All participants provided written informed consent.

**Fig. 1:**
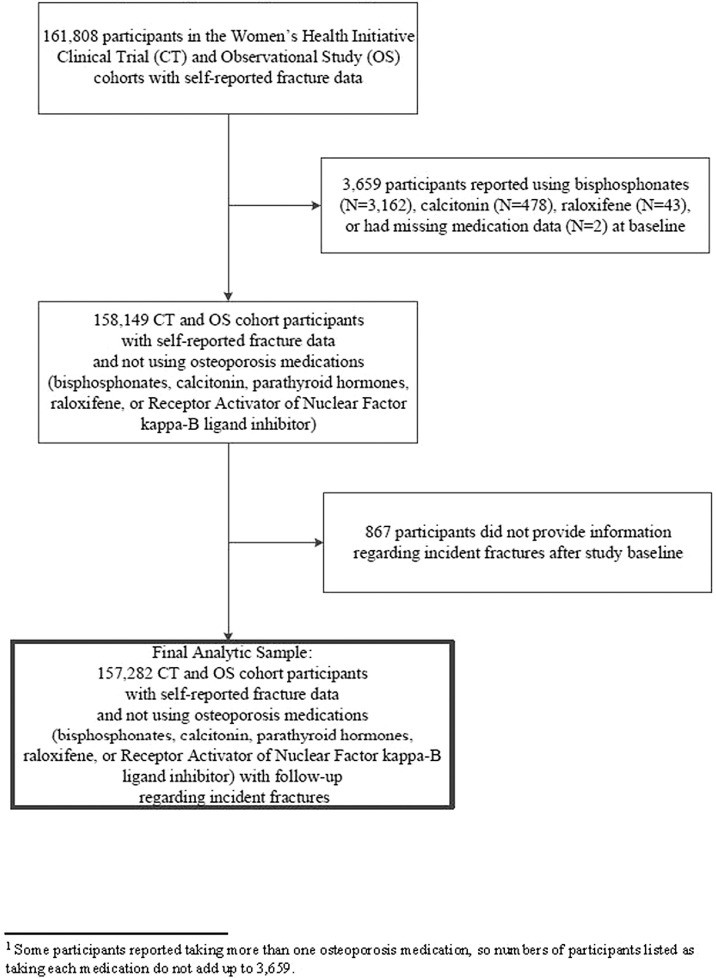
Analytic sample flow (STROBE algorithm).

Of the 161,808 participants of the WHI-OS and WHI-CTs, we excluded data from participants who reported using bisphosphonates (*n* = 3162), calcitonin (*n* = 478), and/or raloxifene (*n* = 43) at baseline, and participants who did not provide information regarding medication use at baseline (*n* = 2) (Fig. 1). No participants were taking parathyroid hormone or denosumab at baseline. We additionally excluded data from participants who did not provide information regarding incident fractures after study baseline (*n* = 867). Therefore, the analytic sample for the current study included 157,282 women; complete covariate information was available for 137,412 participants.

### 2.2 Fracture ascertainment

Incident fractures were initially self-reported (semiannually in WHI-CT, annually in WHI-OS). All fractures were self-reported, with the exception of hip fractures, which were confirmed by study physicians using medical records. Participants were asked “Has a doctor told you for the first time that you have a new broken, crushed, or fractured bone?” Response choices included: hip, upper leg (not hip), pelvis, knee (patella), lower leg or ankle, foot (not toe), tailbone (coccyx), spine or back (vertebra), lower arm or wrist, hand (not finger), elbow, upper arm or shoulder, or other.

The following fractures were excluded: ribs, chest/sternum, skull/face, fingers, toes, cervical vertebrae.

For the current analyses, we classified fractures as lower arm or wrist, upper arm or shoulder, upper leg (not hip), knee (patella), lower leg or ankle, spine, and hip or pelvis. We also examined “any clinical fracture”, defined as fracture of the upper leg (not hip), hip or pelvis, knee (patella), lower leg or ankle, foot (not toe), tailbone (coccyx), spine (vertebra), lower arm or wrist, hand (not finger, elbow, or upper arm or shoulder.

### 2.3 Other variables

Using baseline questionnaires, we collected information regarding age, race/ethnicity, education, smoking, alcohol intake, number of falls in the past 12 months, medication use (including bisphosphonates, calcitonin raloxifene, denosumab, parathyroid hormone, and estrogen), calcium and vitamin D supplement use, and medical history (including diabetes mellitus and fracture prior to WHI study enrollment). Physical activity level was assessed using a validated physical activity questionnaire [11], [12], [13]. Physical function was assessed using the RAND SF-36 physical function score [14,15]. Information about dietary calcium and vitamin D was obtained by food frequency questionnaire [16].

### 2.4 Statistical analysis

Baseline characteristics were described by incident fracture status. Annualized fracture rates were calculated for incident lower arm or wrist, upper arm or shoulder, hip or pelvis, vertebral, upper leg (not hip), knee, and lower leg or ankle fractures and classified by 5-year age groups at screening and race/ethnicity. Incident fractures include the first of each fracture by site or grouping (without previous fracture elsewhere during study follow-up period). Follow-up time for participants without a fracture event was censored at the time of death, the end of study follow-up, or the time of loss to follow-up. Cox proportional hazards models with an initial fracture included as a time-varying exposure were used to examine associations between incident initial fracture and subsequent fracture by anatomical location. In the first set of models, we adjusted the models for WHI clinical trial (trial participation and specific treatment assignment), self-initiated hormone therapy use at study baseline, age, race/ethnicity, and BMI. In subsequent models, we additionally adjusted for education level, smoking status, physical activity (total metabolic equivalent of task h/wk), dietary and supplemental calcium and vitamin D intake, number of falls in the past 12 months, alcohol intake, physical function score, and use of medication harmful to bone health. Subsequently, we stratified the results of the above regression models according to baseline age (50–59, 60–69, 70–79 years-old) and tested for statistical interaction by age. Participants with missing information regarding covariates were excluded from the corresponding Cox models, resulting in a sample size of 137,412 in the fully adjusted models.

Based on *a priori* review of the literature, we performed interaction tests to determine whether associations between initial fracture and subsequent fracture varied by race/ethnicity (excluding American Indian/Alaskan Native and unknown racial/ethnic categories because of insufficient numbers with initial and subsequent fractures) using two groupings of initial fracture sites: upper extremity (initial lower arm/wrist, upper arm/shoulder), or lower extremity (upper leg, knee, lower leg/ankle, or hip fracture).

We performed a sensitivity analyses by accounting for competing risk of all-cause mortality in the Cox proportional hazards models. An additional sensitivity analysis examined associations between initial fracture and any subsequent clinical fracture among menopausal hormone therapy users only.

All analyses were performed using SAS for Windows, v· 9·4 (SAS Institute Inc., Cary, NC, USA).

### 2.5 Role of the funding source

The funding source did not have a role in the writing of the manuscript. CJC had full access to all the data in the study and final responsibility for the decision to submit for publication.

## 3 Results

### 3.1 Baseline characteristics of participants

Mean (SD) participant age was 63·1 (7·2) years and mean (SD) BMI was 28·0 (5·9) kg/m^2^. Eighty-three percent of participants self-identified as White; 25,348 (16%) participants self-identified as non-White. Mean follow-up duration was 15·4 years (SD 6·2, median 18·5, interquartile range 9·0–20·9 years). 47,126 participants (30%) experienced incident fracture

Compared with women who did not experience incident fracture, women with incident fracture were slightly older (*p*-value <0·0001 for difference across age categories), were more likely to be White (*p*-value <0·0001 across race/ethnicity categories), were less likely to be using hormone therapy at baseline (*p*-value = 0·001), were slightly more likely to have experienced falls during the 12 months prior to baseline (*p*-value <0·0001), and were more likely to report a prevalent fracture at study baseline (*p*-value <0·0001) (Table 1).

**Table 1:** Baseline characteristics of participants by incident fracture status^a^.

	Incident Fracture^b^				
	No (*N* = 110,156)		Yes (N 47,126)		
	N	Mean (SD) or%	N	Mean (SD) or%	*P*-value
Age at screening, years	110,156	62.9 (7.2)	47,126	63.8 (7.2)	<0.0001
50–59	38,572	35.0	14,204	30.1	
60–69	48,864	44.4	21,657	46.0	
70–79	22,720	20.6	11,265	23.9	
Race/ethnicity					<0.0001
Non-Hispanic White	87,385	79.3	42,352	89.9	
Non-Hispanic Black/African American	12,275	11.1	2137	4.5	
Hispanic/Latina	5074	4.6	1164	2.5	
American Indian/Alaskan Native	522	0.5	178	0.4	
Asian/Pacific Islander	3264	3.0	734	1.6	
Unknown	1636	1.5	561	1.2	
Education, College degree or higher	41,644	38.1	19,808	42.3	<0.0001
Smoking, current	7970	7.3	2924	6.3	<0.0001
Alcohol drinks per week ≥7	12,218	11.2	6023	12.9	<0.0001
Total daily calcium intake (diet + supplements)	106,611	1164 (720)	45,956	1222 (741)	<0.0001
Total daily vitamin D intake (diet + supplements)	106,611	364 (275)	45,956	382 (280)	<0.0001
Body mass index, kg/m^2^					<0.0001
<25	37,077	34.0	17,049	36.5	
25 - <30	37,637	34.5	16,624	35.6	
>=30	34,471	31.6	13,040	27.9	
Energy expenditure from physical activity, MET hrs/wk	105,182	12.3 (13.8)	44,695	12.7 (13.6)	<0.0001
Physical functioning score^c^	107,921	81.3 (20.1)	46,375	80.9 (19.7)	0.003
Current hormone therapy use at baseline^d^	52,621	47.8	22,089	46.9	0.001
Number of falls in last 12 months at baseline ≥2	11,797	11.2	6957	15.4	<0.0001
History of treated diabetes	4955	4.5	2036	4.3	0.11
History of fracture at age 55+^e^	11,051	13.2	7938	22.2	<0.0001
Use of medication harmful to bone health^f^	3539	3.2	1884	4.0	<0.0001
Estrogen + Progestin Hormone Trial arm^g^					<0.0001
Placebo	5351	47.8	2610	50.7	
Active	5839	52.2	2537	49.3	
Estrogen-Alone Hormone Trial arm^‡‡^					0.001
Placebo	3677	49.7	1667	52.4	
Active	3716	20.3	1517	47.6	
Diet Modification trial arm^‡‡^					0.0004
Control	19,821	40.4	8935	39.2	
Intervention	13,448	59.6	5770	60.8	
Calcium and Vitamin D trial arm^‡‡^					0.01
Control	12,134	50.2	5786	49.7	
Intervention	12,255	49.8	5721	50.3	
Observational Study participant	63,865	58.0	26,491	56.2	<0.0001

a Excluding participants with history of osteoporosis medication use at baseline.

b Any incident clinical fracture, including fractures of the upper leg (not hip), pelvis, knee (patella), lower leg or ankle, foot (not toe), tailbone (coccyx), spine or back (vertebra), lower arm or wrist, hand (not finger), elbow, upper arm or shoulder and hip.

c Assessed using the RAND SF-36 survey.

d Includes self-reported use at baseline and Hormone Therapy (HT) trial active arm participants.

e Excludes participants who were <55 years-old at baseline.

f Includes anticonvulsants, antiestrogens, tamoxifen, antineoplastics, antidepressants, proton pump inhibitors, glucocorticoids, thiazolidinediones and thiazolidinedione-biguanide combinations.

g Denominator only includes corresponding trial participants.

### 3.2 Incident fracture rates

Annualized (unadjusted) incident fracture rates are presented according to age group (Table 2). For each type of fracture other than lower leg/ankle fracture, unadjusted fracture rates were higher in older age groups than younger age groups.

**Table 2:** Incident initial fracture (annualized%) by fracture type and by age group^a^.

	Lower Arm/Wrist		Upper arm or shoulder		Upper Leg		Knee		Lower Leg/Ankle		Hip/Pelvis^b^		Vertebra	
	N	(Ann.%)	N	(Ann.%)	N	(Ann.%)	N	(Ann.%)	N	(Ann.%)	N	(Ann.%)	N	(Ann.%)
Age group at baseline (years)														
50–54	1084	(0·31%)	487	(0·13%)	122	(0·03%)	335	(0·09%)	1196	(0·34%)	199	(0·05%)	487	(0·13%)
55–59	1859	(0·36%)	924	(0·18%)	211	(0·04%)	514	(0·10%)	1708	(0·33%)	467	(0·09%)	950	(0·18%)
60–64	2235	(0·40%)	1148	(0·20%)	345	(0·06%)	681	(0·12%)	1841	(0·33%)	860	(0·15%)	1381	(0·24%)
65–69	2446	(0·50%)	1347	(0·27%)	365	(0·07%)	643	(0·13%)	1547	(0·31%)	1327	(0·26%)	1764	(0·35%)
70–74	1586	(0·52%)	966	(0·31%)	275	(0·09%)	416	(0·13%)	940	(0·30%)	1369	(0·44%)	1382	(0·45%)
75–79	663	(0·59%)	383	(0·34%)	92	(0·08%)	176	(0·15%)	312	(0·27%)	887	(0·79%)	587	(0·52%)

a Ann.: annualized.

b Hip fracture was not adjudicated for Self-Report Cohort (SRC) participants during Extension Study 2. Incident events during extension 2 only include pelvis fracture for SRC participants.

### 3.3 Associations between initial fracture and subsequent fracture

**Fig. 2:**
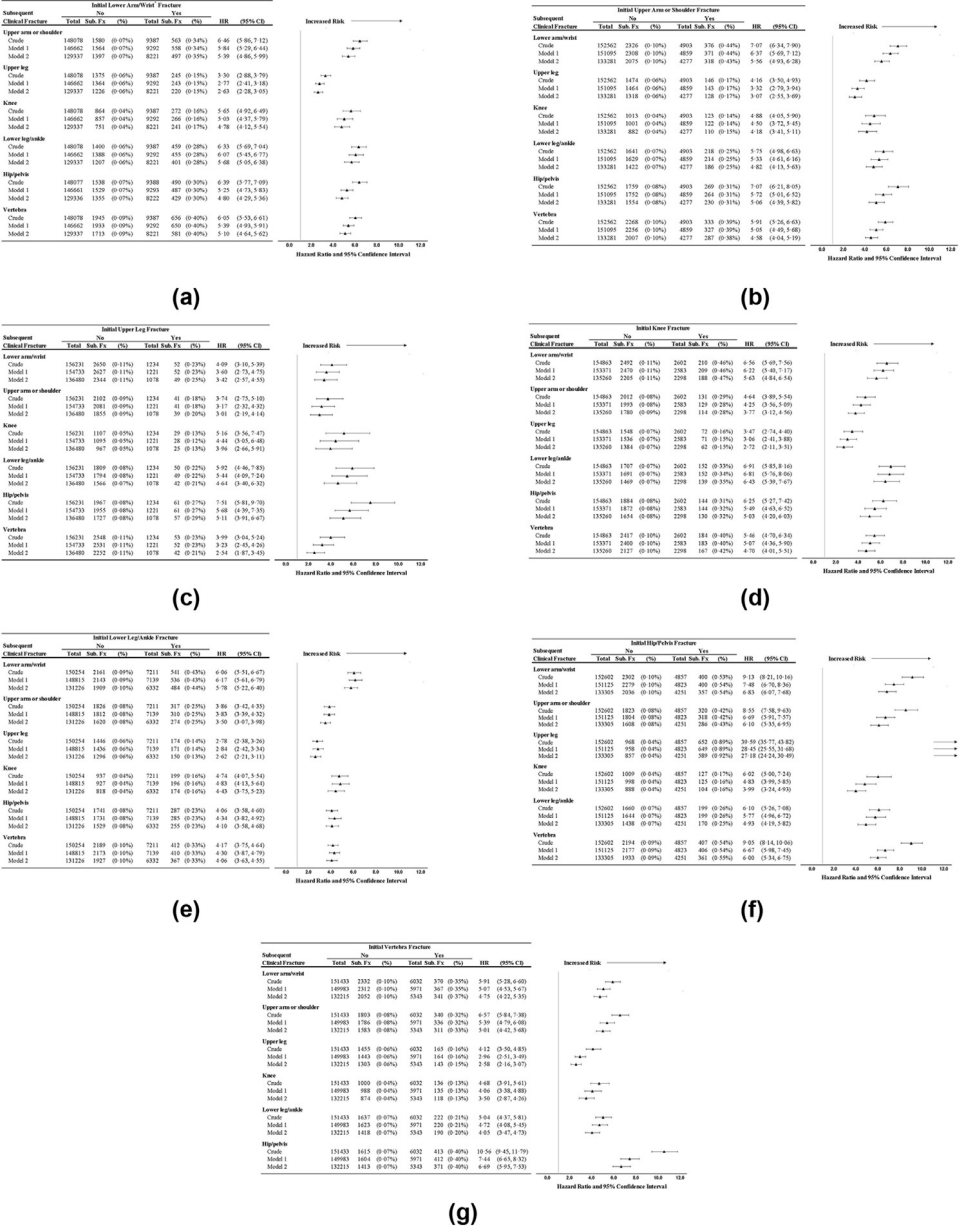
Associations between initial fracture and subsequent fracture by site. Fx: fracture; Sub: subsequent; Ann: Annualized; HR: hazard ratio; CI: confidence interval Model 1 is adjusted for each of the clinical trial (Estrogen + Progestin, Estrogen-alone, Dietary Modification and Calcium + Vitamin D [time-dependent]) randomization arms, age, race/ethnicity, BMI and current hormone use at randomization (WHI Hormone Therapy (HT) trial active randomization arm or current hormone use for non-HT participants). Model 2 is adjusted for covariates in model 1 plus education, smoking status, total metabolic equivalent of task h/wk, total dietary + supplemental calcium intake, total dietary + supplemental vitamin D intake, number of falls, alcohol intake and physical function score. Fig. 2a. Associations between incident lower arm/wrist fracture and subsequent fracture by site. Figure 2b. Associations between incident upper arm or shoulder fracture and subsequent fracture by site. Fig. 2c. Associations between incident upper leg fracture and subsequent fracture by site. Fig. 2d Associations between incident knee fracture and subsequent fracture by site. Fig. 2e. Associations between incident lower leg/ankle fracture and subsequent fracture by site. Fig. 2f. Associations between incident hip/pelvis fracture and subsequent fracture by site. Fig. 2 g. Associations between incident vertebral fracture and subsequent fracture by site.

Adjusted associations between incident fracture and subsequent fracture are shown in Fig. 2a-2g. Incident lower arm/wrist fracture was associated with significantly elevated risks of subsequent fractures at each fracture location examined: upper arm/shoulder, upper leg, knee, lower leg/ankle, hip/pelvis, and vertebrae, with hazard ratios (HRs) ranging from 3·30–6·46 (Fig. 2a). The magnitudes of associations were slightly attenuated after full adjustment for covariates, but all associations remained statistically significant. In models adjusted for age, race/ethnicity, BMI, current estrogen use, and clinical trial intervention group, the most pronounced risk of subsequent fracture after initial incident lower arm/wrist fracture was for subsequent lower leg/ankle fracture, which was 5·68-fold higher (95% confidence internal [CI] 5·05–6·38) among women with, compared to women without, initial lower arm/wrist fracture.

Similar significant associations between incident fractures and increased risk of subsequent fractures were apparent for initial upper arm or shoulder fracture (e.g. adjusted HR [aHR] 5·06 for subsequent hip or pelvis fracture, 95% CI 4·39–5·82, Fig. 2b), initial upper leg fracture (aHR 5·11 for subsequent hip or pelvis fracture, 95% CI 3·91–6·67, Fig. 2c), for initial knee fracture (Fig. 2d), for initial lower leg/ankle fracture (Fig. 2e), for initial hip/pelvis fracture (Fig. 2f), and initial vertebral fracture (Fig. 2g). Initial hip or pelvis fracture was associated with 27-fold higher risk (aHR 27·18) of subsequent upper leg (non-hip) fracture (CI 24·24–30·49).

### 3.4 Associations between initial fracture and subsequent fracture stratified by age at the time of initial fracture and by race/ethnicity

**Fig. 3:**
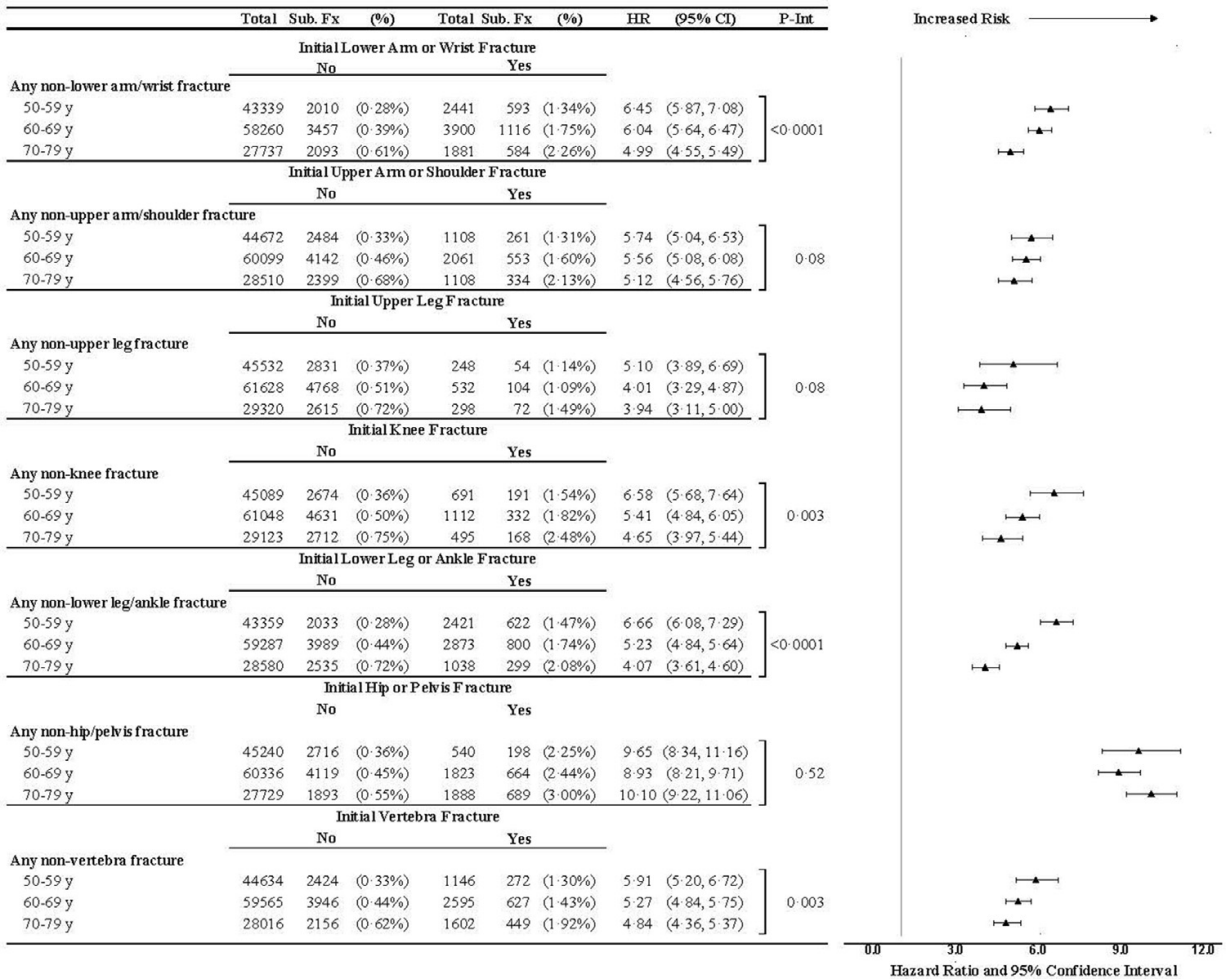
Associations between incident fracture and any subsequent clinical fracture by initial fracture site and age at screening. Fx: fracture; Sub: subsequent; Ann: Annualized; HR: hazard ratio; CI: confidence interval.

We examined the risk of subsequent fracture following initial fracture by age at the time of initial fracture (Fig. 3). Thirty-four percent (1755/5109) of women who experienced initial hip or pelvis fracture experienced a subsequent non-hip fracture. Within each age group, and after each type of initial fracture, the risk of subsequent fracture was significantly higher among women with initial fracture than among women without initial fracture. For example, after an initial lower arm/wrist fracture, the risk of subsequent non-lower arm/wrist fracture was significantly higher in each age group (aHR 6·45 7·56, 95% CI 5·87–7·08 for women 50–59 years-old; aHR 6·04, 95% CI 5·64–6·47 for women 60–69 years-old; aHR 4·99 95% CI 4·55–5·49 for women 70–79 years-old). The HRs were highest for the younger women (50 to 59 years-old) and lowest for the oldest women (70 to 79 years-old). The risk of subsequent fracture after initial fracture varied by age (*P*_interaction_ ranging from <0·0001 to 0·08) for initial lower arm/wrist fracture, initial upper arm/shoulder fracture, initial upper leg, initial knee fracture, initial lower leg fracture, and initial vertebral fracture. In contrast, the risk of any subsequent (non-hip/pelvis) fracture after initial hip/pelvis fracture did not vary significantly by age group (*p*_interaction_ = 0·52), and the point estimate was also higher among older than younger women.

Associations between initial fracture and subsequent fracture varied significantly by race/ethnicity (Table 3) (*p*_interaction_ values ranging from <0·0001 to 0·002). The risk of any subsequent fracture after initial lower extremity fracture ranged from 9-fold to 14-fold higher among non-Hispanic Black women, Hispanic/Latina, and Asian/Pacific Islander women versus 7-fold higher among non-Hispanic White women. A similar pattern was observed for initial upper extremity fracture.

**Table 3:** Associations between incident upper and lower extremity fracture and any subsequent clinical fracture^a^ by initial fracture site and race/ethnicity^b^.

	Total N	Sub. Fx	Ann.%	Total N	Sub. Fx	Ann.%	HR^c^	(95% CI)	P Int
	**Initial Upper Extremity Fracture**								
**Subsequent Clinical Fracture**	**No**			**Yes**					
Any non-lower arm/wrist fracture									<0.0001
Non-Hispanic White	103,772	5473	(0.34%)	11,222	2736	(1.47%)	6.20	(5.92, 6.50)	
Non-Hispanic Black/African American	11,245	126	(0.08%)	426	55	(0.81%)	13.37	(9.61, 18.59)	
Hispanic/Latina	4541	91	(0.16%)	299	49	(1.03%)	8.85	(6.11, 12.81)	
Asian/Pacific Islander	3405	63	(0.14%)	194	46	(1.52%)	17.13	(11.26, 26.08)	
	**Initial Lower Extremity Fracture**								
	**No**			**Yes**					
Any non-upper arm/shoulder fracture									0.002
Non-Hispanic White	102,686	4497	(0.28%)	12,306	2869	(1.45%)	7.48	(7.13, 7.85)	
Non-Hispanic Black/African American	10,988	94	(0.06%)	683	72	(0.67%)	14.32	(10.37, 19.77)	
Hispanic/Latina	4552	114	(0.20%)	288	54	(1.18%)	8.93	(6.34, 12.57)	
Asian/Pacific Islander	3391	57	(0.12%)	208	31	(0.90%)	10.64	(6.69, 16.94)	

a Clinical fracture was defined as fracture of upper leg (not hip), hip or pelvis, knee (patella), lower leg or ankle, foot (not toe), tailbone (coccyx), spine (vertebra), lower arm or wrist, hand (not finger), elbow, upper arm or shoulder.

b Fx: fracture; Sub: subsequent; Ann: Annualized; HR: hazard ratio; CI: confidence interval.

c Hazard ratios (HRs) and 95% confidence intervals (CIs) are adjusted for each of the clinical trial (Estrogen + Progestin, Estrogen-alone, Dietary Modification and Calcium + Vitamin D [time-dependent]) randomization arms, age, BMI, current hormone use at randomization (WHI Hormone Therapy (HT) trial active randomization arm or current hormone use for non-HT participants), education, smoking status, total metabolic equivalent of task h/wk, total dietary + supplemental calcium intake, total dietary + supplemental vitamin D intake, number of falls, alcohol intake and physical function score.

### 3.5 Sensitivity analyses: competing mortality and HT use

In a sensitivity analysis, we explored the potential influence of competing mortality on our results. (Older participants had higher mortality, and therefore lower duration of follow-up time in which to experience a subsequent fracture, yielding a lower-appearing risk of fracture among the oldest women.) However, when we examined the Cox proportional hazards models that additionally accounted for competing mortality, we found that the results did not meaningfully differ from those of the main analyses (Supplemental Table 1).

Results of analyses restricted to participants using HT at baseline or any time during follow-up were very similar to results in the entire analytic sample (Supplemental Table 2).

## 4 Discussion

In this large, prospective cohort of women across the U.S., after adjustment for age, race/ethnicity, BMI, hormone therapy use, and other covariates, every type of initial fracture, including lower arm or wrist, upper arm or shoulder, upper leg, knee, lower leg or ankle, hip or pelvis, and spine, was associated with significantly increased risk of subsequent fracture. Women who experienced initial hip or pelvis fracture were at extremely high risk-a 27-fold higher- risk-of subsequent upper leg (non-hip) fracture. Thirty-four percent of participants who had an initial hip or pelvis fracture had a subsequent non-hip fracture. The findings that knee fracture has the same prognostic value for subsequent fracture as hip or wrist fracture is a novel key finding, as knee fracture is generally not considered “osteoporotic”. Moreover, the risk of subsequent fracture after initial lower arm or wrist fracture, initial upper arm or shoulder fracture, initial knee fracture, initial lower leg or ankle fracture, initial hip or pelvis fracture and spine fracture was significantly higher even among the youngest women aged 50–59 years. We also found important racial/ethnic differences in the associations between initial and subsequent fracture, with higher adjusted hazard ratios among non-White women.

To our knowledge, no previous prospective study has reported detailed patterns of subsequent fracture locations after initial fracture according to age strata among women in the U.S. However, our results are generally consistent with those of a few previous reports with a more limited range of initial fracture sites, more limited participant age range, and/or short duration of follow-up. The National Osteoporosis Risk Assessment study (3-year follow-up) focused only on initial wrist fractures, and found that associations between initial wrist fracture and any future fracture were significantly increased both in women aged < 65 years and in women aged ≥ 65 years; associations did not significantly differ by age group [17]. In that study, the risk for future fracture after initial wrist fracture after adjustment for covariates was increased 2·5- to 3-fold. Similarly, in the Norwegian Tromso Study (follow-up duration 15 years), initial hip, shoulder, and wrist fractures were each associated with increased risk of subsequent nonvertebral fracture in all age groups (50–59, 60–69, 70–79, ≥80 years), with no statistically significant interaction by age group [18]. Finally, in a study of Medicare fee-for-service beneficiaries, there was no significant difference in time to second fracture by age group (66–74, 75–84, 85+ years) [19]. However, in contrast to our study, the previous study did not include women under age 66 years, and follow-up was limited to one year.

Our results have important potential clinical implications. First, the fact that numerous anatomical locations of initial fracture are associated with higher risk of subsequent fracture suggests that guidelines may need to be reassessed. Some clinical guidelines consider previous “fragility fracture” to be diagnostic of osteoporosis [20,21]. (The World Health Organization defines fragility fracture as a fracture resulting from minimal trauma, such as a fall from a standing height) [22]. Other clinical guidelines consider hip or vertebral fractures in the absence of major trauma to be diagnostic of osteoporosis, but do not consider other fracture types to be diagnostic of osteoporosis [23]. On the topic of treatment, some current guidelines recommend that women with hip fracture or vertebral fracture initiate pharmacologic therapy (regardless of bone density level), but women with other types of fracture would not automatically qualify for pharmacologic treatment unless they had bone density in the osteoporotic range [23]. Other treatment guidelines recommend treatment for any type of previous fragility fracture [20], and still other guidelines recommend pharmacologic treatment of persons with hip or spine fragility fractures, “or possibly distal forearm” fracture if they have low bone mass [21]. It will be important to determine whether existing risk calculators can be adapted (or new calculators developed) to help refine decision-making to determine which of the women with fractures other than hip or vertebral fractures should be treated. Also, clinicians should counsel women that these other types of fractures- at sites other than hip and vertebrae- are associated with elevated risk of future fracture, including future hip and pelvic fractures, which are especially linked with increased morbidity, mortality, and loss of independence. Our findings will inform Fracture Liaison Service Programs, which are specifically targeted to preventing subsequent fractures after an initial fracture. Future research should develop and test interventions targeted to decrease fracture risk in women with fractures other than hip or vertebral fractures.

Also notable in the current study is that subsequent fracture risk varied statistically significantly with age, with a pattern of higher point estimates among the youngest women (aged 50–59). Clinicians should be aware that initial fractures of any type in women aged 50–59 years should trigger counseling regarding increased subsequent fracture risk. When we examined statistical models that accounted for the higher mortality of older participants (i.e. lower duration of follow-up), the results did not meaningfully differ from those of the main analysis. These findings are perhaps not surprising because there is not an obvious physiological reason why an initial fracture should be differentially predictive of subsequent fracture in younger than older women. Moreover, although the 95% CIs were overlapping, there were marked differences in risk of subsequent fracture by race/ethnicity, with higher aHRs among Hispanic/Latina, Asian/Pacific Islander, and Black women than among non-Hispanic White women. To our knowledge, these findings have not been well-described in previously-published studies and the reasons for these racial/ethnic patterns are not immediately apparent, but it may be due to more White women initiating therapy following initial fracture than non-White women. There is evidence that osteoporosis treatment is under-diagnosed and under-treated among Black women [24]. These findings highlight potential racial/ethnic disparities in fracture risk that should be an important target for future research.

Our study has potential limitations. First, except for hip fractures, fractures were self-reported in this study. However, in a prior WHI study, agreement between self-report and medical record review was 71% overall, 78% for hip fractures, and 81% for forearm/wrist fractures [25]. The exception was spine fractures, for which agreement was lower (51%). Second, we did not have information regarding rib fracture, which may have resulted in conservative estimates (underestimates) of true associations between initial fracture and subsequent fracture. Third, although we adjusted analyses for potential confounders, residual confounding is possible. Fourth, BMD was measured in a subset of women in WHI (the participants at 4 of the clinical sites) so we could not test whether these associations were independent of BMD. Nevertheless, most women in the WHI Bone Substudy were not osteoporotic by BMD. Finally, we only captured the first initial fracture at each anatomic location. For example, if a participant had a lower arm/wrist fracture, a subsequent lower arm/wrist fracture would not be captured. As a result, our results may underestimate (be conservative estimates of) the associations between initial fracture and subsequent fracture. Strengths of our study include large numbers of participants throughout the U.S., including younger postmenopausal women, prospective follow-up, the assessment of a broader range of initial fracture types than those of previous studies, and detailed information regarding osteoporosis risk factors.

In this large cohort of well-characterized postmenopausal women, we found that all types of fractures, including non-hip and non-vertebral fractures, are associated with increased risk of subsequent fracture, including subsequent hip fracture. Moreover, increased risk of subsequent fracture after initial fracture was apparent for all age groups. For each fracture location, even younger women in their 50′s who experience an initial fracture have a significantly higher risk of subsequent fracture. Future studies should examine potential explanations for the finding that non-White women had higher risk of subsequent fracture after initial fracture than White women. Our results indicate that aggressive follow-up of postmenopausal women who experience initial fracture is indicated. Our results will inform counselling, future guidelines, and the design of intervention trials regarding the selection of appropriate candidates for pharmacotherapy.

## Author contributions

Study design: CJC, RPH, JAC

Data collection: AL, JAC, JWW

Data analysis: RPH

Data interpretation: CJC, RPH, AL, JAR, JWW, KCJ, MS, KLS, JCW, TG, JAC

Manuscript drafting: CJC

Critical review of manuscript for content: CJC, RPH, AL, JAR, JWW, KCJ, MS, KLS, JCW, TRG, JAC

## Funding

The WHI program is funded by the National Heart, Lung, and Blood Institute, National Institutes of Health, U.S. Department of Health and Human Services through contracts HHSN268201600018C,HHSN268201600001C, HHSN268201600002C, HHSN268201600003C, and HHSN268201600004C.

## Data sharing statement

The Women's Health Initiative Study data are publicly-available on the National Heart, Lung, and Blood Institute Biologic Specimen and Data Repository Information Coordinating Center (https://biolincc.nhlbi.nih.gov/studies/).

## Declaration of Competing Interest

TG reports grants from National Heart, Lung, and Blood Institute, during the conduct of the study.

RH reports grants from NHLBI, during the conduct of the study.

KJ reports grants from NIH, during the conduct of the study.

AL reports grants from National Institutes of Health, NHLBI, during the conduct of the study.

KS reports grants from Merck & Co., outside the submitted work.

JW-W reports grants from NHLBI, during the conduct of the study.

MS reports grants from American Cancer Society, grants from The Centers for Disease Control and Prevention, grants from Florida Department of Health, outside the submitted work.

The other authors have nothing to disclose.

## Appendix Supplementary materials

Image, application 1

Image, application 2
