# Supplementary material for: After the initial fracture in postmenopausal women, where do subsequent fractures occur?
Source: eClinicalMedicine. 2021 May 5;35:100826. doi: 10.1016/j.eclinm.2021.100826 (PMC8176125; doi:10.1016/j.eclinm.2021.100826)
Supplement: Supplementary file 1 [file mmc1.docx]

**Supplemental Material**

**After the Initial Fracture in Postmenopausal Women: Where do Subsequent Fractures Occur?**

Carolyn J. Crandall, MD, MS, Rebecca P. Hunt, MS, Andrea Z. LaCroix, PhD, John A. Robbins, MD, MHS, Jean Wactawski-Wende, PhD, Karen C. Johnson, MD, MPH, Maryam Sattari, MD, MS, Katie L. Stone, PhD, Julie C. Weitlauf, PhD, Tanya R. Gure, MD, Jane A. Cauley, DrPH

| **Supplemental Table 1. Associations between incident fracture and any subsequent clinical fracture by initial fracture site and age at screening in the presence of competing risk of all-cause mortality^[[1]](#footnote-1)^** | | | | | | | | | |
| --- | --- | --- | --- | --- | --- | --- | --- | --- | --- |
|  | **Total N** | **Sub. Fx** | **Ann. %** | **Total N** | **Sub. Fx** | **Ann. %** | **HR^[[2]](#footnote-2)^** | **(95% CI)** | **P**  **Int** |
|  | **Initial Lower Arm or Wrist Fracture** | | | | | |  |  |  |
| **Subsequent Clinical Fracture** | **No** | | | **Yes** | | |  |  |  |
| Any non-lower arm/wrist fracture |  |  |  |  |  |  |  |  | 0·0003 |
| 50 – 59 | 43339 | 2010 | (0·28%) | 2441 | 593 | (1·34%) | 6·62 | (5·99, 7·31) |  |
| 60 – 69 | 58260 | 3457 | (0·39%) | 3900 | 1116 | (1·75%) | 6·34 | (5·89, 6·82) |  |
| 70 – 79 | 27737 | 2093 | (0·61%) | 1881 | 584 | (2·26%) | 5·47 | (4·96, 6·03) |  |
|  | **Initial Upper Arm or Shoulder Fracture** | | | | | |  |  |  |
|  | **No** | | | **Yes** | | |  |  |  |
| Any non-upper arm/shoulder fracture |  |  |  |  |  |  |  |  | 0·01 |
| 50 – 59 | 44672 | 2484 | (0·33%) | 1108 | 261 | (1·31%) | 5·74 | (5·01, 6·58) |  |
| 60 – 69 | 60099 | 4142 | (0·46%) | 2061 | 553 | (1·60%) | 5·75 | (5·23, 6·33) |  |
| 70 – 79 | 28510 | 2399 | (0·68%) | 1108 | 334 | (2·13%) | 5·50 | (4·86, 6·21) |  |
|  | **Initial Upper Leg Fracture** | | | | | |  |  |  |
|  | **No** | | | **Yes** | | |  |  |  |
| Any non-upper leg fracture |  |  |  |  |  |  |  |  | 0·04 |
| 50 – 59 | 45532 | 2831 | (0·37%) | 248 | 54 | (1·14%) | 4·93 | (3·71, 6·56) |  |
| 60 – 69 | 61628 | 4768 | (0·51%) | 532 | 104 | (1·09%) | 4·03 | (3·28, 4·95) |  |
| 70 – 79 | 29320 | 2615 | (0·72%) | 298 | 72 | (1·49%) | 4·60 | (3·57, 5·93) |  |
|  | **Initial Knee Fracture** | | | | | |  |  |  |
|  | **No** | | | **Yes** | | |  |  |  |
| Any non-knee fracture |  |  |  |  |  |  |  |  | 0·001 |
| 50 – 59 | 45089 | 2674 | (0·36%) | 691 | 191 | (1·54%) | 6·76 | (5·77, 7·92) |  |
| 60 – 69 | 61048 | 4631 | (0·50%) | 1112 | 332 | (1·82%) | 5·78 | (5·13, 6·52) |  |
| 70 – 79 | 29123 | 2712 | (0·75%) | 495 | 168 | (2·48%) | 5·11 | (4·32, 6·05) |  |
|  | **Initial Lower Leg or Ankle Fracture** | | | | | |  |  |  |
|  | **No** | | | **Yes** | | |  |  |  |
| Any non-lower leg/ankle fracture |  |  |  |  |  |  |  |  | 0·01 |
| 50 – 59 | 43359 | 2033 | (0·28%) | 2421 | 622 | (1·47%) | 6·80 | (6·18, 7·49) |  |
| 60 – 69 | 59287 | 3989 | (0·44%) | 2873 | 800 | (1·74%) | 5·36 | (4·93, 5·82) |  |
| 70 – 79 | 28580 | 2535 | (0·72%) | 1038 | 299 | (2·08%) | 4·51 | (3·96, 5·13) |  |
|  | **Initial Hip or Pelvis Fracture** | | | | | |  |  |  |
|  | **No** | | | **Yes** | | |  |  |  |
| Any non-hip/pelvis fracture |  |  |  |  |  |  |  |  | 0·0004 |
| 50 – 59 | 45240 | 2716 | (0·36%) | 540 | 198 | (2·25%) | 8·99 | (7·59, 10·65) |  |
| 60 – 69 | 60336 | 4119 | (0·45%) | 1823 | 664 | (2·44%) | 8·23 | (7·47, 9·06) |  |
| 70 – 79 | 27729 | 1893 | (0·55%) | 1888 | 689 | (3·00%) | 9·25 | (8·31, 10·30) |  |
|  | **Initial Vertebral Fracture** | | | | | |  |  |  |
|  | **No** | | | **Yes** | | |  |  |  |
| Any vertebral fracture |  |  |  |  |  |  |  |  | 0.001 |
| 50 – 59 | 44634 | 2424 | (0·33%) | 1146 | 272 | (1·30%) | 5·76 | (5·05, 6·58) |  |
| 60 – 69 | 59565 | 3946 | (0·44%) | 2595 | 627 | (1·43%) | 5·19 | (4·74, 5·69) |  |
| 70 – 79 | 28016 | 2156 | (0·62%) | 1602 | 449 | (1·92%) | 5·08 | (4·56, 5·67) |  |

**Supplemental Table 2. Associations between incident fracture and any subsequent clinical fracture by initial fracture site^[[3]](#footnote-3)^ among the total sample and among Menopausal Hormone Therapy (MHT) users^[[4]](#footnote-4)^ only**

|  | **Total N** | **Sub. Fx** | **Ann. %** | **Total N** | **Sub. Fx** | **Ann. %** | **HR^[[5]](#footnote-5)^** | **(95% CI)** |
| --- | --- | --- | --- | --- | --- | --- | --- | --- |
|  | **Initial Lower Arm or Wrist Fracture** | | | | | |  |  |
| **Subsequent Clinical Fracture** | **No** | | | **Yes** | | |  |  |
| Any non-lower arm/wrist fracture |  |  |  |  |  |  |  |  |
| Full sample | 128748 | 7560 | (0·39%) | 8664 | 2293 | (1·63%) | 5·76 | (5·49, 6·04) |
| MHT users | 73929 | 4328 | (0·37%) | 4619 | 1178 | (1·51%) | 5·90 | (5·52, 6·30) |
|  | **Initial Upper Arm or Shoulder Fracture** | | | | | |  |  |
|  | **No** | | | **Yes** | | |  |  |
| Any non-upper arm/shoulder fracture |  |  |  |  |  |  |  |  |
| Full sample | 132829 | 9025 | (0·45%) | 4583 | 1148 | (1·53%) | 5·35 | (5·03, 5·70) |
| MHT users | 76025 | 5078 | (0·42%) | 2520 | 621 | (1·45%) | 5·57 | (5·11, 6·06) |
|  | **Initial Upper Leg Fracture** | | | | | |  |  |
|  | **No** | | | **Yes** | | |  |  |
| Any non-upper leg fracture |  |  |  |  |  |  |  |  |
| Full sample | 136187 | 10214 | (0·50%) | 1225 | 230 | (1·07%) | 4·10 | (3·59, 4·67) |
| MHT users | 77855 | 5714 | (0·47%) | 693 | 125 | (0·99%) | 4·17 | (3·48, 4·98) |
|  | **Initial Knee Fracture** | | | | | |  |  |
|  | **No** | | | **Yes** | | |  |  |
| Any non-knee fracture |  |  |  |  |  |  |  |  |
| Full sample | 134970 | 10017 | (0·49%) | 2442 | 691 | (1·74%) | 5·43 | (5·02, 5·87) |
| MHT users | 77174 | 5560 | (0·46%) | 1369 | 386 | (1·68%) | 5·82 | (5·24, 6·45) |
|  | **Initial Lower Leg or Ankle Fracture** | | | | | |  |  |
|  | **No** | | | **Yes** | | |  |  |
| Any non-lower leg/ankle fracture |  |  |  |  |  |  |  |  |
| Full sample | 130784 | 8557 | (0·44%) | 6628 | 1721 | (1·61%) | 5·38 | (5·10, 5·67) |
| MHT users | 74680 | 4713 | (0·40%) | 3865 | 994 | (1·54%) | 5·70 | (5·32, 6·11) |
|  | **Initial Hip or Pelvis Fracture** | | | | | |  |  |
|  | **No** | | | **Yes** | | |  |  |
| Any non-hip/pelvis fracture |  |  |  |  |  |  |  |  |
| Full sample | 132942 | 8728 | (0·43%) | 4470 | 1551 | (2·48%) | 9·18 | (8·68, 9·71) |
| MHT users | 76152 | 4963 | (0·41%) | 2394 | 813 | (2·33%) | 8·71 | (8·06, 9·42) |
|  | **Initial Vertebra Fracture** | | | | | |  |  |
|  | **No** | | | **Yes** | | |  |  |
| Any non-vertebra fracture |  |  |  |  |  |  |  |  |
| Full sample | 131612 | 8526 | (0·43%) | 5800 | 1348 | (1·41%) | 5·15 | (4·86, 5·47) |
| MHT users | 75167 | 4721 | (0·40%) | 3378 | 759 | (1·33%) | 5·26 | (4·86, 5·69) |

1. Fx: fracture; Sub: subsequent; Ann: Annualized; HR: hazard ratio; CI: confidence interval; Int: interaction [↑](#footnote-ref-1)
2. Hazard ratios (HRs) and 95% confidence intervals (CIs) in the presence of competing risk of all-cause mortality are adjusted for each of the clinical trial (Estrogen + Progestin, Estrogen-alone, DM and CaD (time-dependent)) randomization arms, race/ethnicity, BMI, current hormone use at randomization (WHI Hormone Therapy (HT) trial active randomization arm or current hormone use for non-HT participants), education, smoking status, total metabolic equivalent of task h/wk, total dietary + supplemental calcium intake, total dietary + supplemental vitamin D intake, number of falls, alcohol intake and physical function score. [↑](#footnote-ref-2)
3. Fx: fracture; Sub: subsequent; Ann: Annualized; HR: hazard ratio; CI: confidence interval [↑](#footnote-ref-3)
4. Includes HT trial participants randomized to an active arm (E-alone or E+P), non-HT trial participants with personal use at baseline and all participants with personal use during follow-up prior to subsequent fracture. [↑](#footnote-ref-4)
5. Hazard ratios (HRs) and 95% confidence intervals (CIs) are adjusted for each of the clinical trial (Estrogen + Progestin, Estrogen-alone, Dietary Modification and Calcium + Vitamin D [time-dependent]) randomization arms, race/ethnicity, BMI, current hormone use at randomization (WHI Hormone Therapy (HT) trial active randomization arm or current hormone use for non-HT participants), education, smoking status, total metabolic equivalent of task h/wk, total dietary + supplemental calcium intake, total dietary + supplemental vitamin D intake, number of falls, alcohol intake and physical function score. [↑](#footnote-ref-5)
