# Supplementary material for: After the initial fracture in postmenopausal women, where do subsequent fractures occur?
Source: eClinicalMedicine. 2021 May 5;35:100826. doi: 10.1016/j.eclinm.2021.100826 (PMC8176125; doi:10.1016/j.eclinm.2021.100826)
Supplement: Supplementary file 2 [file mmc2.docx]

**List of WHI Investigators**

Program Office:

(National Heart, Lung, and Blood Institute, Bethesda, Maryland) Jacques Rossouw, Shari Ludlam, Joan McGowan, Leslie Ford, and Nancy Geller

Clinical Coordinating Center: (Fred Hutchinson Cancer Research Center, Seattle, WA) Garnet

Anderson, Ross Prentice, Andrea LaCroix, and Charles Kooperberg

Investigators and Academic Centers: (Brigham and Women's Hospital, Harvard Medical

School, Boston, MA) JoAnn E. Manson; (MedStar Health Research Institute/Howard University, Washington, DC) Barbara V. Howard; (Stanford Prevention Research Center, Stanford, CA)

Marcia L. Stefanick; (The Ohio State University, Columbus, OH) Rebecca Jackson; (University

of Arizona, Tucson/Phoenix, AZ) Cynthia A. Thomson; (University at Buffalo, Buffalo, NY) Jean Wactawski-Wende; (University of Florida, Gainesville/Jacksonville, FL) Marian Limacher; (University of Iowa, Iowa City/Davenport, IA) Jennifer Robinson; (University of Pittsburgh, Pittsburgh, PA) Lewis Kuller; (Wake Forest University School of Medicine, Winston-Salem, NC) Sally Shumaker; (University of Nevada, Reno, NV) Robert Brunner
